# Visual and patient-reported outcomes of a diffractive trifocal intraocular lens in highly myopic eyes: a prospective multicenter study

**DOI:** 10.1186/s40662-023-00336-3

**Published:** 2023-04-06

**Authors:** Jiaqi Meng, Yanwen Fang, Jingcai Lian, Xu Chen, Jing Zhou, Wenwen He, Keke Zhang, Fan Yang, Yi Lu, Xiangjia Zhu

**Affiliations:** 1 Eye Institute and Department of Ophthalmology, Eye & ENT Hospital, Fudan University, 83 Fenyang Road, Xuhui District, Shanghai, 200031 China; 2 NHC Key Laboratory of Myopia (Fudan University), Key Laboratory of Myopia, Chinese Academy of Medical Sciences, Shanghai, 200031 China; 3 Shanghai Key Laboratory of Visual Impairment and Restoration, Shanghai, 200031 China; 4 Shanghai Xinshijie Zhongxing Eye Hospital, Shanghai, China; 5 Department of Ophthalmology, Shanghai Aier Eye Hospital, Shanghai, China; 6 Shanghai Bright Eye Hospital, Shanghai, China; 7 State Key Laboratory of Medical Neurobiology, Fudan University, Shanghai, 200032 China

**Keywords:** Trifocal intraocular lens, High myopia, Multicenter, Axial length, Cataract

## Abstract

**Background:**

To investigate the visual and patient-reported outcomes of a diffractive trifocal intraocular lens (IOL) in highly myopic eyes.

**Methods:**

Patients with planned cataract removal by phacoemulsification and implantation of a trifocal IOL (AT LISA tri 839MP) were enrolled in the prospective, multicenter cohort study. Patients were allocated into three groups according to their axial length (AL): control group, AL < 26 mm; high myopia group, AL 26–28 mm; extreme myopia group, AL ≥ 28 mm. At 3 months post-surgery, data for 456 eyes of 456 patients were collected, including visual acuity, defocus curve, contrast sensitivity (CS), visual quality, spectacle independence, and overall satisfaction.

**Results:**

After surgery, the uncorrected distance visual acuity improved from 0.59 ± 0.41 to 0.06 ± 0.12 logMAR (*P* < 0.001). In all three groups, about 60% of eyes achieved uncorrected near and intermediate visual acuity of 0.10 logMAR or better, but significantly fewer eyes in the extreme myopia group achieved uncorrected distance visual acuity of 0.10 logMAR or better (*P* < 0.05). Defocus curves revealed that the visual acuity was significantly worse in the extreme myopia group than others at 0.00, − 0.50, and − 2.00 diopters (*P* < 0.05). CS did not differ between the control and high myopia groups but was significantly lower in the extreme myopia group at 3 cycles per degree. The extreme myopia group also had greater higher-order aberrations and coma, lower modulation transfer functions and VF-14 scores, more glare and halos, worse spectacle independence at far distance, and consequently lower patient satisfaction than others (all *P* < 0.05).

**Conclusions:**

In eyes with a high degree of myopia (AL < 28 mm), trifocal IOLs have been shown to provide similar visual outcomes to those in non-myopic eyes. However, in extremely myopic eyes, acceptable results may be obtained with trifocal IOLs, but a reduced level of uncorrected distance vision is expected.

**Supplementary Information:**

The online version contains supplementary material available at 10.1186/s40662-023-00336-3.

## Background

To meet the increasing visual demands of patients, multifocal intraocular lenses (IOLs) are widely implanted during cataract surgery to achieve spectacle independence [1, 2]. Bifocal IOLs can improve unaided near and distance visual acuity, but concerns remain regarding spectacle dependence at intermediate distance and postoperative dysphotopsia. Therefore, trifocal IOLs, which provide better intermediate visual acuity, have recently drawn much attention [3–5].

In the last two decades, the prevalence of high myopia has increased dramatically worldwide [6]. Patients with high myopia often develop cataracts at a younger age (30–50 years) [7] when they still have a strong need for near and intermediate vision. As these patients have typically worn glasses for a long time, they often desire to be free of glasses after cataract surgery. Multifocal IOLs, particularly trifocal IOLs, may allow these patients to achieve spectacle independence as other options such as corneal refractive surgery and intraocular collamer lenses are not suitable for eyes with cataracts.

However, there have been few studies on the effectiveness of trifocal IOLs in highly myopic eyes, partly because trifocal IOLs are mostly used in European countries where the prevalence of high myopia is relatively low [6]. One potential reason for the lack of studies on trifocal IOLs in highly myopic eyes is that surgeons may be hesitant to use these lenses due to the anatomic complexity of highly myopic eyes, the uncertainty of surgical outcomes, and the higher risk of retinal detachment in this population [8–12]. Additionally, trifocal IOLs are more expensive and may not be a preferred option for some surgeons. In light of these factors, the purpose of this study was to evaluate the visual and patient-reported outcomes of a diffractive trifocal IOL (AT LISA tri 839MP, Carl Zeiss AG) in highly myopic eyes in a large prospective multicenter cohort.

## Methods

This prospective multicenter cohort study was initiated by the Eye and Ear, Nose, Throat (EENT) Hospital of Fudan University, Shanghai, China, between October 1st, 2019 and October 1st, 2021, in collaboration with three other medical institutions: Xinshijie Zhongxing Eye Hospital, Shanghai Aier Eye Hospital, and Shanghai Bright Eye Hospital in Shanghai, China. The protocol was approved by the institutional review board of EENT Hospital of Fudan University (No. 2020109), and the study was conducted in accordance with the tenets of the Declaration of Helsinki. Written informed consent was obtained from all patients.

### Patients

**Fig. 1:**
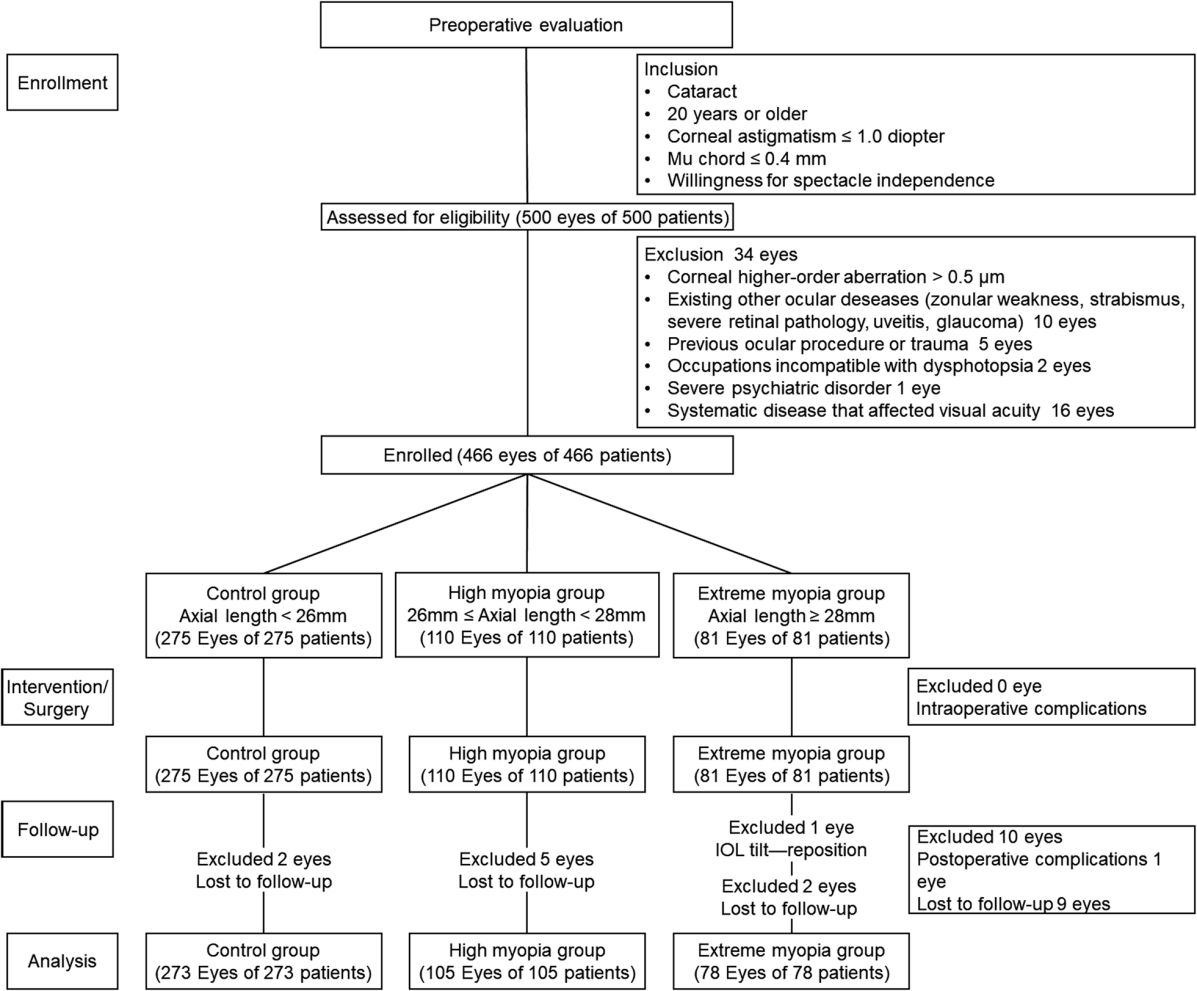
Flowchart of patient enrollment and follow-up

The inclusion criteria were cataract patients aged ≥ 20 years, with a corneal astigmatism of ≤ 1.0 diopter (D), a mu chord of ≤ 0.4 mm, and a willingness for spectacle independence. The exclusion criteria included high corneal higher-order aberration (HOA) > 0.5 μm, zonular weakness, strabismus, severe retinal pathology, uveitis, glaucoma, previous ocular procedure or trauma, occupation incompatible with dysphotopsia, severe psychiatric disorder, or any systematic disease that affected visual acuity, such as diabetes. According to the definition of high myopia and previous studies [13, 14], the eyes were subdivided based on their axial length (AL): control group (AL < 26 mm), high myopia group (AL 26–28 mm), and extreme myopia group (AL ≥ 28 mm). Patients with intraoperative or postoperative complications or who were lost to follow-up were excluded from the analysis. Finally, a total of 456 eyes of 456 patients were available for analysis. Figure 1 shows the flowchart of study enrollment.

### Preoperative assessment

Before surgery, all patients underwent complete ophthalmic examinations, which included visual acuity, slit-lamp examination, corneal topography (Pentacam HR, OCULUS Optikgerate, Wetzlar, Germany), AL measurement (IOLMaster700, Carl Zeiss AG, Oberkochen, Germany), and a fundus examination. The IOL power required was calculated with the Barrett Universal II formula (Lens Factor + 1.62 or A Constant 118.5) and was selected with a target of emmetropia [14–16]. No minus power IOL was used.

### Surgical procedure

Surgery was performed by an experienced surgeon in each medical institution (JL, JZ, XC, and YL, respectively) using standard procedures. After making a 2.6 mm clear corneal incision temporally, a continuous curvilinear capsulorhexis with a diameter of approximately 5.5 mm, was created. After cataract removal by phacoemulsification, the anterior chamber was filled with an ophthalmic viscosurgical device. The trifocal IOL (AT LISA tri 839MP, Carl Zeiss AG) was then implanted in the capsular bag with the injection technique recommended by the manufacturer. The IOL power ranges from 0.0 D to + 32.0 D with 0.5 D increments (www.zeiss.com). The ophthalmic viscosurgical device was thoroughly removed before the incision was hydrated. The position of the IOL was checked before the completion of surgery. No intraoperative complication was observed in the study.

### Postoperative follow-up

All patients were reviewed at three months after surgery at each medical institution, in accordance with the study protocol. Uncorrected near visual acuity (UNVA; logarithms of the minimal angle of resolution, logMAR) at 40 cm, uncorrected intermediate visual acuity (UIVA; logMAR) at 80 cm, uncorrected distance visual acuity (UDVA; logMAR) at 4 m, and corrected distance visual acuity (CDVA; logMAR) were evaluated using an Early Treatment Diabetic Retinopathy Study (ETDRS) chart (Wehen Vision Technology Co. Ltd, Guangzhou, China) under 85 cd/m^2^, and manifest refraction were also recorded. The prediction error was defined as the difference between the postoperative refraction and the predicted refraction. To determine the monocular defocus curves, the patients were fitted with their best-corrected distance refraction, and their visual acuity measured between + 1.00 D and − 4.00 D in defocus increments of 0.50 D. Measurements were started in negative lenses to prevent memorization.

Monocular contrast sensitivity (CS) was assessed with best distance correction using the Optec6500 (Stereo Optical Co., Inc., Chicago, IL, USA). Assessments were made at spatial frequencies of 1.5, 3, 6, 12, and 18 cycles per degree (cpd), with or without glare, under photopic (background luminance of 85 cd/m^2^) or mesopic conditions (background luminance of 3 cd/m^2^). Patches not seen were accounted with the value of the last patch, and all patients saw at least one patch. The log base 10 contrast sensitivity values were used for statistical analysis according to previous studies [17, 18].

Ocular and intraocular HOAs (root mean square [RMS]) and the modulation transfer function (MTF) were measured with an OPD Scan III aberrometer (Nidek Co, Ltd., Gamagori, Japan) [4, 19, 20] after dilating the pupil and recorded for 6.0 mm and 4.0 mm pupil diameters. Photopic and mesopic pupil diameter were recorded by OPD Scan III aberrometer before pupil dilation.

To assess their subjective visual quality, all patients completed a visual function questionnaire (VF-14). VF-14 is scaled from 0 to 100, with a higher score indicating better subjective visual function. The patients were also asked about symptoms of dysphotopsia, including glare, halos, and starbursts. Four-point response scales were used to assess each symptom: from “never” to “always” for the frequency, from “none” to “severe” for the severity, and from “not bothered at all” to “bothered very much” for bothersomeness. Information on spectacle independence and overall satisfaction was collected by asking the patients whether they used spectacles for near vision (reading, sewing), intermediate vision (computer work, housework), or far vision (watching television, driving) and whether they were satisfied with the visual outcomes after surgery.

### Statistical analysis

Means ± standard deviations were used to describe continuous variables, and percentages were used to describe the distributions of categorical variables. A paired *t-*test was used to compare preoperative and postoperative visual acuity. One-way analysis of variance (ANOVA) with Tukey’s post hoc test was used to compare continuous variables among groups. The χ^2^ test was used to compare categorical variables among groups. All statistical analyses were performed with SPSS software (ver. 22.0, IBM, Chicago, IL, USA) and missing data were not imputed. All tests were two-tailed, and the level of statistical significance was set to *P* < 0.05.

## Results

Adverse events are included in Fig. 1. At one month after surgery, a patient with significant IOL tilt received IOL reposition, who were not included in the final analysis. At three months post-surgery, no patient lost two lines of CDVA and 1.1% (5/456) did not achieve CDVA of 0.3 logMAR.

### Patient characteristics

The mean age of all the participants was 59.4 ± 11.1 years, and 56.6% were female. The mean AL of the highly myopic eyes was 27.78 ± 1.39 mm (median, 27.61 mm; range, 26.01–31.39 mm). Table 1 shows the characteristics of the three groups. There were no differences in the baseline features among the three groups (all *P* > 0.05) other than AL and calculated IOL power.

**Table 1:** Patient characteristics

Parameters	Control group (n = 273)	High myopia group (n = 105)	Extreme myopia group (n = 78)	*P* value
Age (years)^a^	60.5 ± 12.0	58.4 ± 9.2	57.7 ± 9.1	0.063
Sex, male/female^b^	119/154	42/63	37/41	0.602
Eye laterality, right/left^b^	141/132	52/53	42/36	0.844
Axial length (mm)^a^	23.73 ± 1.08	26.83 ± 0.63	29.09 ± 0.87	< 0.001
Preoperative corneal power (D)^a^	43.09 ± 1.17	43.06 ± 1.41	42.82 ± 0.96	0.211
Preoperative corneal astigmatism (D)^a^	0.64 ± 0.37	0.69 ± 0.39	0.73 ± 0.42	0.193
Mu chord (mm)^a^	0.20 ± 0.11	0.21 ± 0.11	0.21 ± 0.11	0.946
Mesopic pupil diameter (mm)^a^	4.66 ± 0.72	4.70 ± 0.65	4.85 ± 0.63	0.111
Photopic pupil diameter (mm)^a^	3.51 ± 0.44	3.50 ± 0.46	3.59 ± 0.41	0.333
Preoperative CDVA (logMAR)^a^	0.58 ± 0.39	0.64 ± 0.37	0.61 ± 0.41	0.375
IOL power (D)^a^	19.8 ± 3.1	11.4 ± 3.8	7.6 ± 4.6	< 0.001

*D* = diopter; *CDVA* = corrected distance visual acuity; *logMAR* = logarithm of the minimal angle of resolution; *IOL* = intraocular lens

^a^One-way ANOVA with Tukey’s post hoc test

^b^χ^2^ test

*P* values less than 0.05 are considered statistically significant

### Visual acuity

**Fig. 2:**
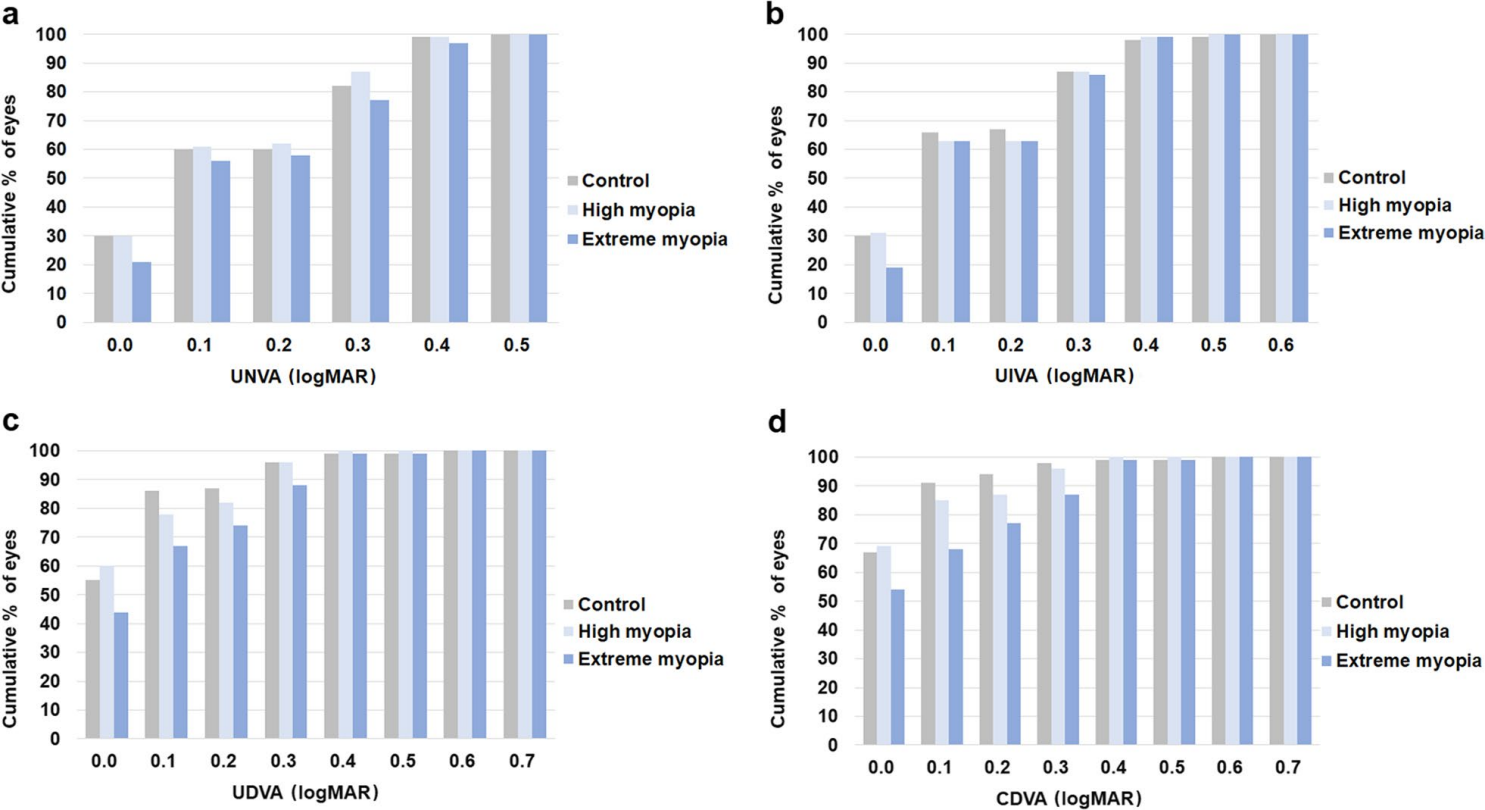
Cumulative monocular visual acuities after implantation of the trifocal intraocular lens. Cumulative monocular UNVA (**a**), monocular UIVA (**b**), monocular UDVA (**c**), and monocular CDVA (**d**) after implantation of the trifocal intraocular lens in the control, high myopia and extreme myopia groups. logMAR, logarithm of the minimum angle of resolution; UNVA, uncorrected near visual acuity; UIVA, uncorrected intermediate visual acuity; UDVA, uncorrected distance visual acuity; CDVA, corrected distance visual acuity

After trifocal IOL implantation, UDVA improved significantly in all patients, from 0.59 ± 0.41 logMAR before surgery to 0.06 ± 0.12 logMAR at three months after surgery (paired *t*-test; *P* < 0.001). In all three groups, about 60% of eyes achieved UNVA and UIVA of 0.10 logMAR or better (Fig. 2). However, the cumulative percentage of eyes with UDVA and CDVA of 0.10 logMAR or better was significantly lower in the extreme myopia group than in the control and high myopia groups in terms of UDVA (67%, 86%, and 78%, respectively; χ^2^ test, *P* = 0.006) and CDVA (68%, 91%, and 85%, respectively; χ^2^ test, *P* < 0.001). Table 2 compares the visual acuities and prediction errors among the three groups after surgery. No differences were detected in visual acuities between the control and high myopia groups (one-way ANOVA with Tukey’s post hoc test, all *P* > 0.05), but the mean UDVA and CDVA were significantly worse in the extreme myopia group than in the control group (one-way ANOVA with Tukey’s post hoc test, both *P* < 0.05).

**Table 2:** Visual acuities and prediction errors among the three groups after trifocal intraocular lens implantation

	Control group (n = 273)	High myopia group (n = 105)	Extreme myopia group (n = 78)	*P* value
UNVA (logMAR)	0.13 ± 0.13	0.11 ± 0.13	0.15 ± 0.13	0.214
UIVA (logMAR)	0.12 ± 0.12	0.12 ± 0.11	0.13 ± 0.10	0.590
UDVA (logMAR)	0.05 ± 0.11	0.06 ± 0.11	0.10 ± 0.14	0.005^a^
CDVA (logMAR)	0.02 ± 0.10	0.03 ± 0.10	0.08 ± 0.13	< 0.001^b^
SE (D)	− 0.18 ± 0.42	− 0.22 ± 0.44	− 0.19 ± 0.57	0.811
Prediction error (D)	− 0.09 ± 0.36	− 0.09 ± 0.33	− 0.13 ± 0.46	0.699

*UNVA* = uncorrected near visual acuity; *logMAR* = logarithm of the minimal angle of resolution; *UIVA* = uncorrected intermediate visual acuity; *UDVA* = uncorrected distance visual acuity; *CDVA* = corrected distance visual acuity; *D* = diopter; *SE* = spherical equivalent

^a^Significant difference between control and extreme myopia groups

^b^Significant difference between extreme myopia group and the other two groups

*P* values less than 0.05 are considered statistically significant

No difference in the spherical equivalent (SE) and prediction error was detected among the groups (one-way ANOVA with Tukey’s post hoc test, *P* > 0.05). The percentage of eyes with SE of more than 1.0 D was significantly higher in the extreme myopia group than in the control group (10.3% vs. 2.2%, respectively; χ^2^ test, *P* = 0.004). The percentage of eyes with a prediction error of within ± 0.5 D did not differ among the control, high myopia, and extreme myopia groups (84%, 86%, and 77%, respectively; χ^2^ test, *P* = 0.224), but the percentage of eyes with a prediction error of more than 1.0 D was significantly higher in the extreme myopia group than in the control group (6% vs. 0%, respectively; χ^2^ test, *P* = 0.003).

### Defocus curves

**Fig. 3:**
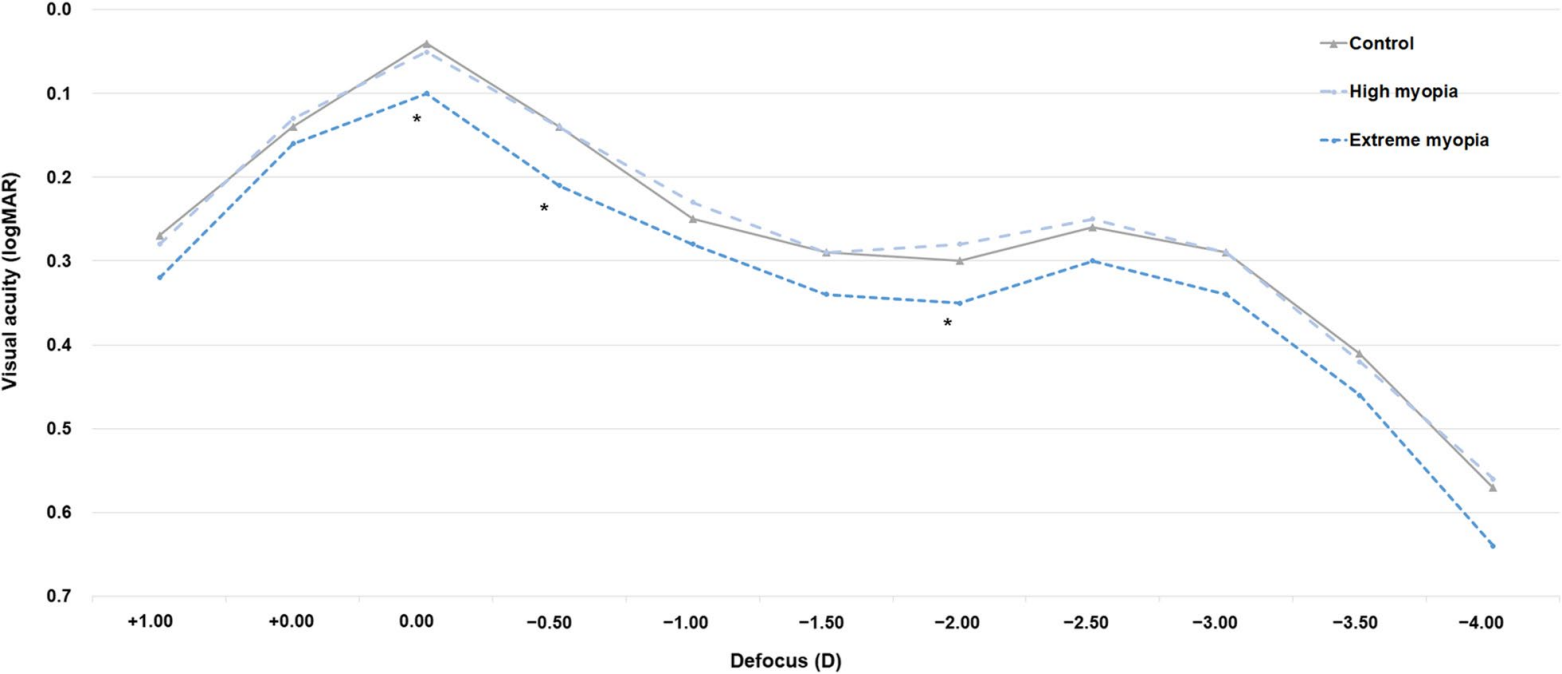
The monocular defocus curves of the trifocal intraocular lens. logMAR, logarithm of the minimum angle of resolution; D, diopter; * Statistically significant (*P* < 0.05)

The monocular defocus curves for the trifocal IOLs are shown in Fig. 3. In each group, there was a peak of maximal visual acuity at the far focus, corresponding to 0.00 D. Another peak was observed at the near focus, corresponding to − 2.50 D. The defocus curves did not differ significantly between the control and high myopia groups, but the extreme myopia group showed significantly worse visual acuity than the other groups at defocus levels of 0.00, − 0.50, and − 2.00 D (one-way ANOVA with Tukey’s post hoc test, *P* = 0.004, 0.007, and 0.034, respectively).

### Contrast sensitivity

**Fig. 4:**
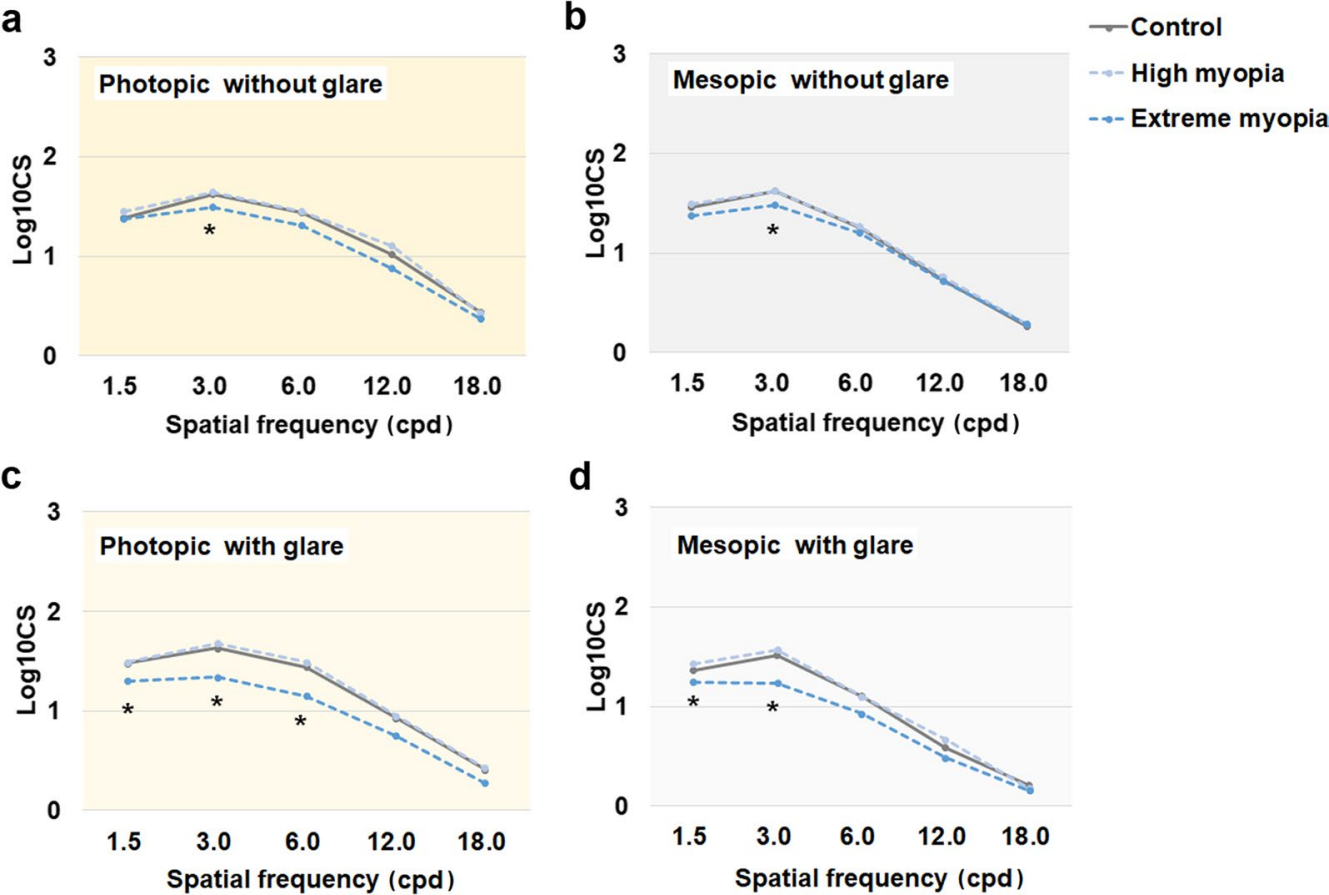
Monocular contrast sensitivity curves after implantation of the trifocal intraocular lens. The monocular contrast sensitivity data at different spatial frequencies were obtained without glare under photopic (**a**) and mesopic (**b**) conditions, or with glare under photopic (**c**) and mesopic (**d**) conditions. CS, contrast sensitivity; cpd, cycles per degree. *Statistically significant (*P* < 0.05)

Monocular contrast sensitivity under photopic and mesopic conditions with or without glare are shown in Fig. 4. Under either photopic or mesopic conditions, CS was the greatest at a spatial frequency of 3 cpd but decreased at higher spatial frequencies. There were no differences in CS between the control and high myopia groups, regardless of the measurement condition (all *P* > 0.05). However, the extreme myopia group showed significantly worse photopic and mesopic CS at 3 cpd without glare compared with the control and high myopia groups (one-way ANOVA with Tukey’s post hoc test, *P* = 0.014 and 0.034, respectively). When measured with glare, the extreme myopia group also showed significantly worse photopic CS at 1.5–6 cpd and significantly worse mesopic CS at 1.5–3 cpd (one-way ANOVA with Tukey’s post hoc test, all *P* < 0.05).

### Objective quality

The HOAs and MTFs of the trifocal IOL in different groups are presented in the Additional file 1). At a pupil diameter of 6 mm, the ocular coma and spherical aberrations were significantly greater in the extreme myopia group than in the control group (one-way ANOVA with Tukey’s post hoc test, *P* = 0.019 and 0.015, respectively). The extreme myopia group also showed significantly greater intraocular HOAs and coma aberration than the other two groups (one-way ANOVA with Tukey’s post hoc test, all *P* < 0.05). At a pupil diameter of 4 mm, the ocular and intraocular HOAs and coma aberrations were significantly greater in the extreme myopia group than in the control group (one-way ANOVA with Tukey’s post hoc test, all *P* < 0.05). The extreme myopia group also displayed significantly lower ocular and intraocular MTFs than the other groups at spatial frequencies of 10, 20, and 30 cpd, for both pupil diameters (one-way ANOVA with Tukey’s post hoc test, all *P* < 0.05).

### Patient reported outcomes

**Fig. 5:**
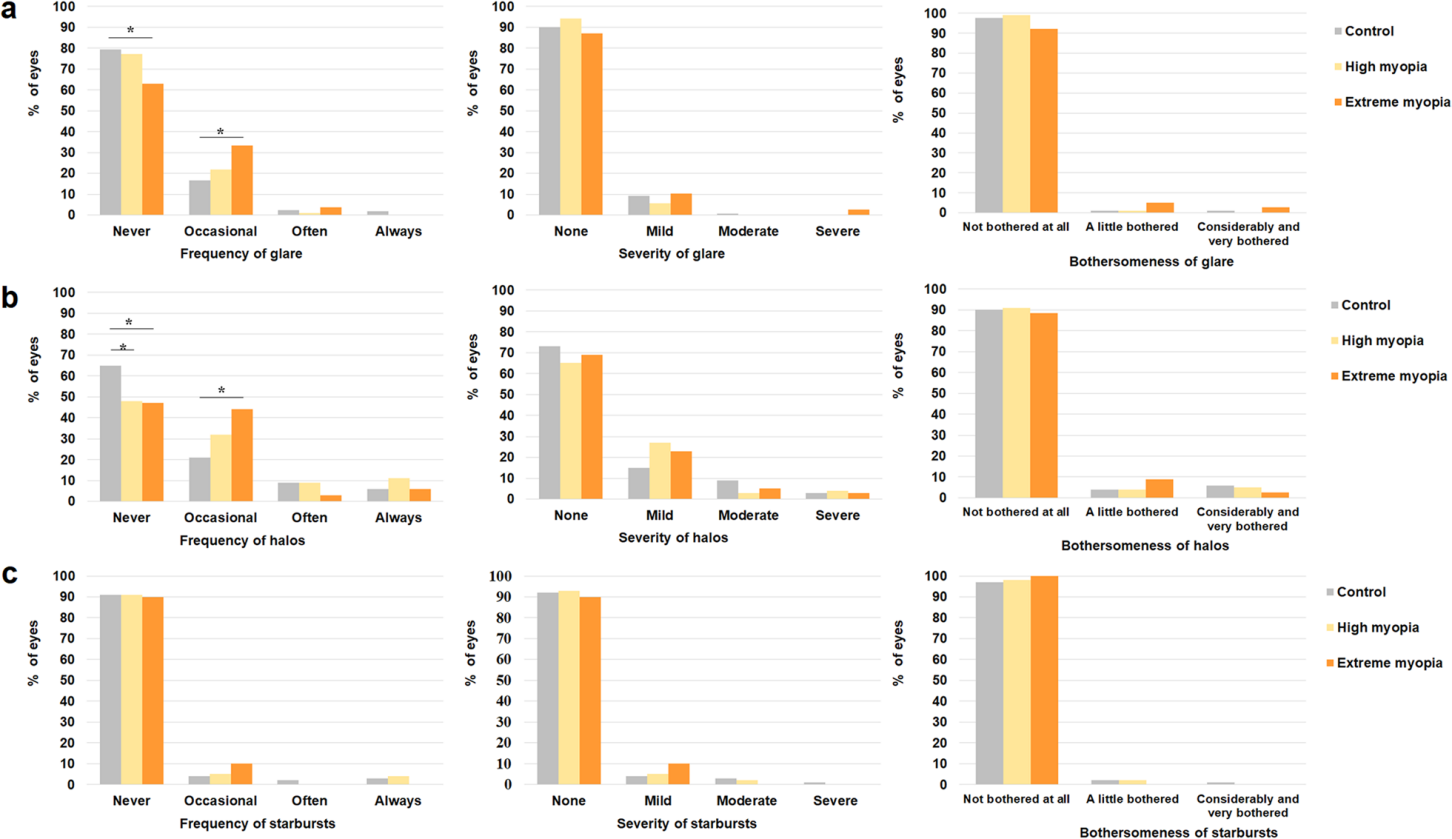
Symptoms of dysphotopsia after implantation of the trifocal intraocular lens. The percentages of different responses to the frequency, severity and bothersomeness of glare (**a**), halos (**b**) and starbursts (**c**) in the control, high myopia and extreme myopia groups. *Statistically significant (*P* < 0.05)

In terms of subjective visual quality, the extreme myopia group had a significantly lower VF-14 score than the other two groups (97.49 ± 5.86, 96.51 ± 8.41, and 94.69 ± 8.21 for the control, high myopia, and extreme myopia groups, respectively; one-way ANOVA with Tukey’s post hoc test, *P* = 0.007). 2.2% (6/273), 1.9% (2/105) and 6.4% (5/78) of the control, high myopia and extreme myopia group had great difficulties driving at night from the VF-14, respectively (χ^2^ test, *P* = 0.115). Glare was more frequent in the extreme myopia group than in the control group (37% vs. 20%, respectively; χ^2^ test, *P* = 0.017), although there was no difference in its severity among the three groups (Fig. 5). Halos were more frequent in the high myopia and extreme myopia groups than in the control group (52%, 53%, and 35%, respectively; χ^2^ test, *P* < 0.001), although there were no differences in the severity or bothersomeness of halos among the groups. The frequency, severity, and bothersomeness of starbursts did not differ among the groups (χ^2^ test, all *P* > 0.05).

Table 3 presents the rates of spectacle independence and patient satisfaction in each group. Overall, spectacle independence did not differ significantly among the groups. The rates of spectacle independence at near and intermediate distances in all groups were better than 90%, but the rate at far distance was significantly lower in the extreme myopia group than in the other groups (χ^2^ test, *P* = 0.043).

**Table 3:** Spectacle independence and patient satisfaction among the three groups after trifocal intraocular lens implantation

	Control group (n = 273)	High myopia group (n = 105)	Extreme myopia group (n = 78)	*P* value
Spectacle independence, n (%)				
Near	246 (90.1%)	98 (93.3%)	71 (91.0%)	0.618
Intermediate	272 (99.6%)	105 (100.0%)	78 (100.0%)	0.715
Far	269 (98.5%)	102 (97.1%)	73 (93.6%)	0.043
Overall	241 (88.3%)	96 (91.4%)	67 (85.9%)	0.491
Satisfaction, n (%)				
Satisfied	250 (91.6%)	102 (97.1%)	68 (87.2%)	0.041
Partly satisfied	17 (6.2%)	3 (2.9%)	8 (10.3%)	0.119
Dissatisfied	6 (2.2%)	0	2 (2.6%)	0.289
Reasons for dissatisfaction				
Spectacle dependence	3 (50%)	–	1 (50%)	–
Poor CS	–	–	1 (50%)	–
Dysphotopsia	3 (50%)	–	–	–

*CS* = contrast sensitivity

*P* values less than 0.05 are considered statistically significant

The rates of patient satisfaction in the control, high myopia, and extreme myopia groups were 91.6%, 97.1%, and 87.2%, respectively (χ^2^ test, *P* = 0.041). Six patients in the control group and two patients in the extreme myopia group felt dissatisfied with the outcome of surgery. Their reasons for dissatisfaction were mainly spectacle dependence for near and far vision, poor CS, and significant symptoms of dysphotopsia. Of the high myopia group who were partly satisfied, one patient showed high refractive error, and two others cannot tolerate dysphotopsia. Of the extreme myopia group who were dissatisfied, one showed high refractive error, and another showed poor CS.

## Discussion

In recent years, trifocal IOLs have become a new option for cataract surgery, with the aim of providing good functional vision at a wide range of distances [4, 21, 22]. With the strong demand for spectacle independence, highly myopic patients with cataract may benefit from the implantation of trifocal IOLs. However, few studies have investigated the efficacy of trifocal IOLs in large cohorts of highly myopic patients. In this large prospective multicenter study, we analyzed the visual and patient-reported outcomes of a diffractive trifocal IOL in highly myopic eyes. We found that in highly myopic eyes with AL < 28 mm, trifocal IOLs provided similar visual outcomes to those of the controls and previous reported long-term studies for the same IOL [23]. However, extremely myopic eyes resulted in lower but acceptable UDVA and only comparable UIVA and UNVA. This was due to the lower predictability of Barrett formula in this range.

The application of multifocal IOLs to highly myopic eyes is challenging [11, 20, 24]. Previous studies have shown that bifocal IOLs in highly myopic eyes can achieve good visual acuity at both near and far distances [10, 25, 26], but several studies have reported worse visual outcomes in eyes with longer ALs [27, 28]. Studies of trifocal IOLs have reported improved intermediate visual acuity with no reduction in near and far vision in emmetropic eyes, but have not addressed high myopia [4, 5]. In fact, most of these studies examined small numbers of patients with a narrow range of ALs, and lacked detailed assessments of visual quality. Therefore, comprehensive evaluations of visual and patient-reported outcomes following implantation of trifocal IOLs in highly myopic eyes is needed. In this study of a large, prospective, multicenter cohort, we found that highly myopic patients with AL < 28 mm experienced good visual outcomes that were similar to the control patients after trifocal IOL implantation, including good near, intermediate, and distance visual acuity and satisfactory objective and subjective visual quality. Thus, our study provides direct, objective, and powerful evidence supporting the use of trifocal IOLs in highly myopic eyes.

The defocus curves revealed that highly myopic eyes with AL < 28 mm achieved good functional vision at near to far distances after surgery. Previous studies of the same trifocal IOL showed that the visual acuity of non-myopic eyes was − 0.08 to 0.08 logMAR at 0.00 D, 0.06 to 0.12 logMAR at − 1.50 D, and 0.02 to 0.16 logMAR at − 2.50 D [29, 30]. Here, the visual acuity of highly myopic eyes with AL < 28 mm was maintained at greater than 0.30 logMAR (the limit of good functional vision) from + 1.00 D to − 3.00 D, which was similar to previous reports [29–31]. This indicates that highly myopic eyes can achieve satisfactory visual acuity during daily activities at different distances, such as reading, computer-related tasks, and driving, which was also supported by the high rate of spectacle independence (approximately 90%) in this group.

Importantly, both the subjective and objective visual quality, despite the limitations of the latest [34], in highly myopic patients with AL < 28 mm were as good as those without high myopia. Contrast sensitivity plays an important role in determining the postoperative visual quality after cataract surgery [11, 33]. In this study, the CS function curves of highly myopic eyes with AL < 28 mm were similar to those of the controls. Moreover, no significant differences in HOA or MTF were detected between the highly myopic eyes with AL < 28 mm and the control eyes. The incidence of glare and starbursts in the highly myopic eyes were 23% and 9%, respectively, which were similar to the values for control eyes and those in previous reports [30, 34]. Notably, halos were more frequently seen in highly myopic eyes, regardless of the AL, but did not usually affect the daily lives of the patients and faded with time.

Another interesting phenomenon was the slightly better patient satisfaction among the highly myopic patients with AL < 28 mm, even compared with patients without high myopia. In this study, the rates of satisfaction were 91.6% and 97.1% in the control and highly myopic patients with AL < 28 mm, respectively, and the rates of dissatisfaction were 2.2% and 0%, respectively. A possible explanation is that the experience of getting rid of heavy glasses that had been worn for a long time offset the highly myopic patients concern about slight “side effects” of the trifocal IOLs. Thus, trifocal IOLs may be a good option for highly myopic patients with cataract, AL < 28 mm, and a relatively healthy fundus, allowing them to achieve spectacle independence.

Despite the satisfying visual and patient-reported outcomes for the highly myopic eyes with AL < 28 mm, the use of trifocal IOLs in extremely long eyes should be approached with caution because the postoperative visual performance is uncertain. Steinwender et al. reported that the UNVA, UIVA, and UDVA of eyes with a trifocal IOL power of 0.0–10.0 D were 0.12, 0.13, and 0.06 logMAR, respectively, in a group of 10 patients [25]. Alfonso et al. reported that the UNVA and UDVA of eyes with a bifocal IOL power of 10.0–15.0 D were 0.16 and 0.02 logMAR, respectively [27]. Previous studies failed to identify differences in these parameters between patients with low and high myopia [27, 28, 35], possibly because their sample sizes were small or the AL ranges were narrow. In this prospective multicenter study, we found that eyes with AL ≥ 28 mm may have worse distance visual acuity and also perform worse in defocus tests than other eyes after the implantation of a trifocal IOL, particularly at 0.00 D, − 0.50 D, and − 2.00 D. The worse distance visual acuity may be related with higher prediction error of IOL calculation and reduced macular sensitivity in these extreme eyes. The sensitivity of the trifocals is between 0.50 to 0.75 D. However, the percentage of eyes with prediction error of more than 1.00 D was significantly higher in the extreme myopia group, suggesting poor prediction accuracy in these eyes. Moreover, the decline of macular sensitivity in high myopes can occur before severe fundus pathology happens, which also affects their visual function [36, 37].

The visual quality achieved with trifocal IOLs in extremely myopic eyes with AL of ≥ 28 mm was worse than that of the other groups. Others have different opinions on whether myopia influences the CS of eyes implanted with multifocal IOLs [27, 28]. Fernandez-Vega et al. [28] found no difference in CS after bifocal IOL implantation between eyes with low to moderate myopia with an IOL power of 15.0–20.5 D and highly myopic eyes with an IOL power of 0.0–14.5 D, whereas Alfonso et al. [27] reported a lower mesopic CS at spatial frequencies of 3–12 cpd in highly myopic eyes than in eyes with low or moderate myopia after the implantation of bifocal IOLs. Our data show that CS tended to be significantly lower in extremely myopic eyes, especially at low spatial frequencies and with glare, and was also lower than the reported results for emmetropic eyes implanted with the same type of IOL and healthy eyes [29, 38, 39]. Extremely myopic patients also displayed increased HOA, reduced MTF, lower VF-14 scores, and poorer satisfaction after trifocal IOL implantation compared with the other groups in our study. Therefore, it is important to inform patients with highly myopic eyes that the visual quality improvement they may experience with trifocal IOL implantation may not meet their expectations.

Here, the reasons for dissatisfaction among patients with extreme myopia, were mainly spectacle dependence and poor CS. One of the major factors affecting visual outcomes may be the unique anatomic characteristics of extremely myopic eyes and the accompanying fundus lesions, such as photoreceptor degeneration, mild myopic macular degeneration and choroidal thinning [36, 37]. With longer AL, the visual function of highly myopic eyes may be worse than that of emmetropic eyes [9]. Even with a thorough fundus examination, contrast sensitivity may be impaired by preclinical pathological changes in highly myopic eyes. Another factor that can affect the visual outcomes of trifocal IOLs in eyes with AL ≥ 28 mm is prediction error. We observed that more than 6% of extremely myopic eyes had a prediction error of over 1.00 D after IOL implantation, which may be due to less accurate preoperative biometry and IOL formulas [40, 41]. Prediction error after multifocal IOL implantation can also cause patient dissatisfaction [42, 43]. While 85.9% of extremely myopic eyes achieved spectacle independence with trifocal IOL implantation, there were also some disadvantages such as worse UDVA, poor CS, more fundus lesions, and a greater prediction error compared to other groups. These potential drawbacks may contribute to uncertainty about the surgical efficacy in these patients and impact patient satisfaction. Therefore, it is important to have appropriate preoperative discussions with patients about the potential risks and benefits of trifocal IOL implantation in extremely myopic eyes.

The main strengths of this study were the prospective multicenter design, the large sample size, the wide range of ALs, and the comprehensive evaluation of visual and patient-reported outcomes of trifocal IOLs in highly myopic eyes. Our findings may help with IOL selection for preoperative communication with highly myopic patients with cataract. Furthermore, the satisfactory performance of trifocal IOLs in highly myopic eyes may benefit from several external factors, including the use of high-resolution optical coherence tomography and super-wide fundus photographs to exclude potential fundus retinopathy [44, 45], the development of optical biometry and next-generation IOL formulas [15, 46, 47], advances in devices to evaluate aberrations and corneal astigmatism, and the improvement of patient management and follow-up. Advances in these technological aspects may also improve the efficacy of trifocal IOLs in extremely long eyes in the future.

One limitation of our study may be that aberrometry results from OPD Scan III in multifocal IOL can be difficult to interpret. Conventional aberrometry, such as Hartmann-Shack wavefront sensor and laser ray tracing aberrometry, cannot accurately represent ocular aberrations of multifocal IOLs, while double-pass techniques or the recently proposed multifocal reconstruction method may provide more accurate estimates of the eye’s image quality [48, 49]. Further study is needed to accurately measure aberrations in multifocal IOLs. Moreover, as large AL is accompanied by inaccurate effective lens position, a capsular tension ring (CTR) is often used to maintain the shape of capsular bags in cases with very low IOL power or zonular weakness, which may affect postoperative refraction. However, no CTR was used in this study, but studies have shown that CTRs do not significantly influence the postoperative IOL position and refractive prediction error.^53,54^ Another limitation of this study is that the questionnaire responses of patients who received trifocal IOL implantation in only one eye may be influenced by the refractive power of the contralateral eye. Additionally, a study with longer follow-up time would be beneficial.

## Conclusions

This large prospective multicenter cohort study found that trifocal IOLs provided similar visual outcomes in highly myopic eyes (AL < 28 mm) as in eyes without high myopia. However, in extremely myopic eyes, acceptable results may be obtained with trifocal IOLs, but a reduced level of uncorrected distance vision is expected due to the higher prediction error in IOL power calculation. Despite this, the implantation of multifocal IOLs in patients with AL > 28 mm is not contraindicated, as long as patients are informed about the potential visual disadvantages before surgery. Further research is needed to assess the long-term safety of trifocal IOLs in this population.

## Supplementary Information

**Additional file 1: Figure S1**. Higher-order aberrations (HOAs) and modulation transfer functions after implantation of the trifocal intraocular lens. Ocular and intraocular HOAs for 6 mm (a, b) and 4 mm (c, d) pupil diameters. Ocular and intraocular modulation transfer functions (MTF) at different spatial frequencies for 6 mm (e, f) and 4 mm (g, h) pupil diameters. RMS, root mean square; cpd, cycles per degree. Error bars represent standard error of the mean. *P < 0.05, **P < 0.01, ***P < 0.001.

## Data Availability

The datasets used and/or analyzed during the current study are available from the corresponding author on reasonable request.

## Abbreviations

IOL: Intraocular lens
D: Diopter
AL: Axial length
Κ: Kappa
UNVA: Uncorrected near visual acuity
logMAR: Logarithm of the minimum angle of resolution
UIVA: Uncorrected intermediate visual acuity
UDVA: Uncorrected distance visual acuity
CDVA: Corrected distance visual acuity
SE: Spherical equivalent
CS: Contrast sensitivity
cpd: Cycles per degree
HOAs: Higher-order aberrations
RMS: Root mean square
MTF: Modulation transfer function
ANOVA: One-way analysis of variance
