# Supplementary figures and images for: Visual and patient-reported outcomes of a diffractive trifocal intraocular lens in highly myopic eyes: a prospective multicenter study
Source: Eye Vis (Lond). 2023 Apr 6;10:19. doi: 10.1186/s40662-023-00336-3 (PMC10077756; doi:10.1186/s40662-023-00336-3)

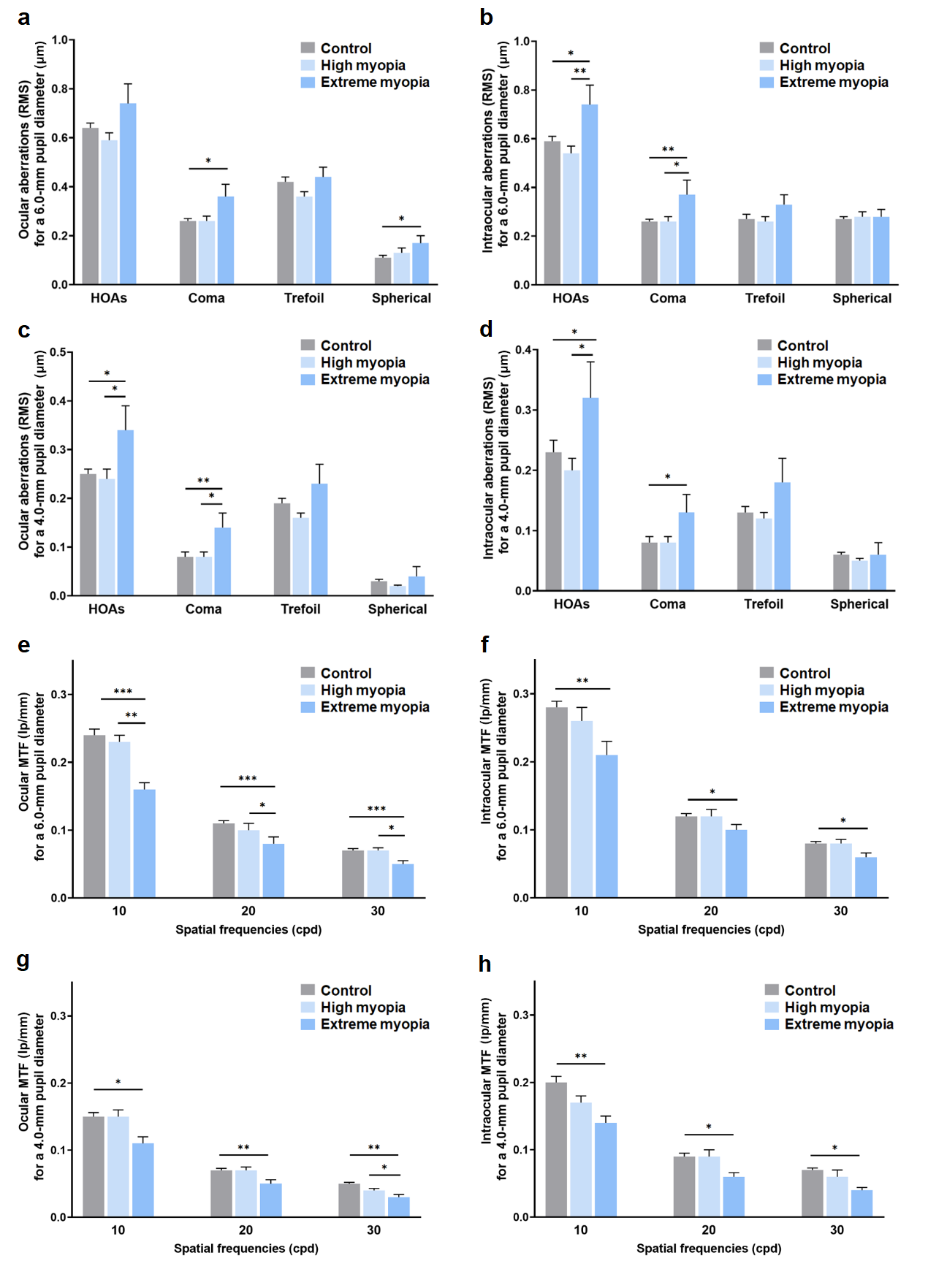

Supplement: Supplementary file 1 — Additional file 1: Figure S1. Higher-order aberrations (HOAs) and modulation transfer functions after implantation of the trifocal intraocular lens. Ocular and intraocular HOAs for 6 mm (a, b) and 4 mm (c, d) pupil diameters. Ocular and intraocular modulation transfer functions (MTF) at different spatial frequencies for 6 mm (e, f) and 4 mm (g, h) pupil diameters. RMS, root mean square; cpd, cycles per degree. Error bars represent standard error of the mean. *P < 0.05, **P < 0.01, ***P < 0.001. [file 40662_2023_336_MOESM1_ESM.tif]
